# Prevalence of Vitamin D Deficiency in Singapore: Its Implications to Cardiovascular Risk Factors

**DOI:** 10.1371/journal.pone.0147616

**Published:** 2016-01-22

**Authors:** Xinyan Bi, Siew Ling Tey, Claudia Leong, Rina Quek, Christiani Jeyakumar Henry

**Affiliations:** 1 Clinical Nutrition Research Centre, Centre for Translational Medicine, 14 Medical Drive #07–02, MD 6 Building, Singapore 117599, Singapore; 2 Singapore Institute for Clinical Sciences, Agency for Science, Technology and Research (A*STAR), Brenner Centre for Molecular Medicine, 30 Medical Drive, Singapore 117609, Singapore; 3 Department of Biochemistry, Yong Loo Lin School of Medicine, National University of Singapore, Singapore 117599, Singapore; University of Leicester, UNITED KINGDOM

## Abstract

**Objective:**

Vitamin D deficiency is a global health challenge and has been linked to type 2 diabetes and other chronic diseases. However, the relationship between vitamin D status, body composition, and cardiovascular risks has not been well characterized in Asian populations. The objectives of this study were to examine the factors associated with the low vitamin D levels in a sunny tropical region and to assess the role of vitamin D status in cardiovascular risk factors.

**Design and Methods:**

This was a cross-sectional study. One hundred and fourteen healthy participants (59 males and 55 females) residing in Singapore took part in this study. Plasma 25OH-D_3_ concentration was measured by using LC-MS/MS. Body fat (%) was measured by using three different techniques including bioelectrical impedance analysis (BIA), BOD POD, and dual-energy X-ray absorptiometry (DEXA). Basic anthropometric measurements, fasting blood glucose (FBG), fasting serum insulin (FSI), and lipid profiles were obtained using standard protocols.

**Results:**

Approximately 42% of the participants were vitamin D deficient (< 20 ng/mL). Vitamin D status was inversely associated with body fat (%), homeostasis model assessment of insulin resistance (HOMA-IR), and total cholesterol/high density lipoprotein (TC/HDL) ratio, while positively associated with lean body mass (LBM) and hand grip strength (HGS).

**Conclusions:**

The high prevalence of vitamin D deficiency in a sunny tropical region reinforces the need to recognize that sunlight alone is not the precursor for optimal vitamin D status. This raises the need to investigate public health measures that will encourage exposure to sunlight without overexposure that is harmful to skin. More importantly, vitamin D deficiency is associated with increased cardiovascular risks, i.e. HOMA-IR, TC/HDL, and LDL/HDL. Future studies should attempt to elucidate the potential mechanisms.

## Introduction

The predominant source of vitamin D in humans is exposure to sunlight [1]. Hence, the observation that people living in the sunny region of the world still suffer from vitamin D deficiency remains an enigma. Anything that interferes with the penetration of ultraviolet irradiation into the skin may affect the vitamin D status [2]. Previous studies have shown that increased skin pigment dramatically reduced vitamin D synthesis [3]. Many other factors, such as age, season, latitude, and kidney function have also been associated with the vitamin D status [4, 5]. In addition, obesity has been suggested as a risk factor for vitamin D deficiency. Due to the fat-soluble properties, vitamin D is readily stored in adipose tissue. Therefore, vitamin D deficiency is usually associated with increased body fat and is prevalent in obese population. For example, obese adults who took vitamin D_2_ supplement and were exposed to UV light had vitamin D levels substantially lower than non-obese counterparts [6].

The classical actions of vitamin D include the regulation of mineral ion homeostasis and bone metabolism. Therefore, vitamin D has been associated primarily with bone health, and it is well known that vitamin D can reduce bone resorption and subsequent bone loss. Prior studies in adults suggested that vitamin D increased bone mineral density (BMD) [7], which in turn associated with decreased osteoporotic fractures [8], and better musculoskeletal function in the lower-extremities of the elderly [9]. However, conflicting results were obtained in young women [10]. In fact, the relation between vitamin D levels and BMD may be complex. It appeared to be varied by race, being weaker in African-American or Hispanic ethnicity than in white populations [11]. Whereas most of the studies focused on Caucasians and African-Americans, studies on Asian, especially Southeast Asia populations are scarce and the association between vitamin D deficiency and body fat, muscle strength, and bone health in this population remains uncertain.

More recently, nonclassical actions of vitamin D have been recognized, e.g. control of cell growth and differentiation, regulation of immune function and endocrine effects, such as insulin resistance, inflammation, renal and muscle function [12–14]. Vitamin D receptor (VDR), which triggers most of vitamin D actions, is widely distributed across almost all the major human organs including heart, brain, livers, bone, kidney, and urinary system, as well as a number of tissues such as immune cells, pancreatic beta cells, cardiomyocytes, endothelial cells, and vascular smooth cells. Through the widely distributed VDR, vitamin D controls vital genes related to bone metabolism, oxidative damage, inflammation, and chronic diseases [15, 16]. Therefore, vitamin D deficiency has been linked to a whole spectrum of diseases including osteoporosis, cancer, diabetes, and cardiovascular and immune disorders [17–20].

Evolving data indicated that vitamin D was capable to influence pancreatic beta-cell proliferation and survival; and hence impaired vitamin D status was associated with higher prevalence and incidence of diabetes [21]. Several previous studies have shown that lower vitamin D status was associated with increased fasting blood glucose (FBG) levels [22, 23] while other studies in Malay adults (mean age of 48.5 y; 42% male) and young Thais have yielded conflicting results [24]; some, but not all, found an association between vitamin D and the risk of diabetes mellitus [25–27]. Additionally, adequate vitamin D status is important for optimal function of cardiovascular system. It has been reported that vitamin D deficiency caused an increase in parathyroid hormone (PTH), which increased insulin resistance and was associated with diabetes, hypertension, inflammation, and increased cardiovascular risks [1]. Epidemiological studies have associated vitamin D deficiency with coronary risk factors and adverse cardiovascular outcomes. The strong association between vitamin D status and cardiometabolic health may contribute to the higher risk of all-cause mortality in vitamin D deficient individuals [28]. However, other studies in very young obese children found no association between vitamin D status and insulin resistance or cardiovascular risks [29]. The above mentioned contradictory results could be attributed to the differences in participant characteristics, e.g. age, gender, race/ethnicity, diet, countries where participants reside, and methods used to measure vitamin D status, e.g. immunoassay, HPLC, and LC-MS/MS.

Taking into account the concomitantly high prevalence of vitamin D deficiency and obesity worldwide, one of the purposes of the current study was to provide an estimate of vitamin D status in population living in a sunny tropical region and to determine the association between vitamin D levels, body fat, muscle strength, and bone health in this population. We also evaluated the effects of vitamin D deficiency on insulin resistance and cardiovascular risks in these healthy adults.

## Material and Methods

### Study design and sample

This study was conducted using a cross-sectional design which was a subsection of a larger study. A total of 114 healthy adults consisting of 59 males and 55 females were recruited from the general public in Singapore through advertisements and posters that were placed on website. To be eligible, participants were required to be Singaporeans or individuals who have resided in Singapore for a minimum of five years, healthy males and females aged between 21 and 100 y. Participants were excluded if they were pregnant or diagnosed with any major diseases. This study was conducted according to the guidelines laid down in the Declaration of Helsinki. The National Healthcare Group Domain Specific Review Board (NHG DSRB, Reference Number: 2013/00783), Singapore approved this study. All participants gave written informed consent.

### Clinical measures

Participants arrived at the laboratory in the morning after a 10 h overnight fast. Two finger prick capillary blood samples were obtained for determining blood glucose concentration using the HemoCue^®^ 201+ RT Glucose analyser (HemoCue Ltd, Dronfield, UK). In addition, a total of 10 mL of venous blood was collected into Vacutainers (Becton Dickinson Diagnostics). Blood samples were separated by centrifugation at 1500 rpm for 10 min at 4°C within 2 h of being drawn and aliquots were stored at -80°C until analysis. Serum insulin (μU/mL) was measured using the immunochemistry analyzer COBAS e411 (Roche, HITACHI, USA). Total cholesterol (TC), high density lipoprotein (HDL), low density lipoprotein (LDL), and triglycerides (TG) were measured by using the immunochemistry analyzer COBAS c311 (Roche, HITACHI, USA).

Standing height was measured to the nearest millimetre by using a stadiometer. Body weight and composition were measured to the nearest 0.1 kg by using an 8-electrode bioelectrical impedance analysis (BIA) device (Tanita BC-418, Tokyo, Japan). The body mass index (BMI) was calculated using weight (kg) divided by the height squared (m^2^). Participants were weighed in light clothing without footwear. Fat mass and percentage body fat were measured by using Bod Pod^TM®^ Body Composition Tracking System (Cosmed, Rome, Italy; software version 5.2.0) and DEXA (QDR 4500A, fan-beam densitometer, software version 8.21; Hologic, Waltham, USA). In addition, bone mineral content (BMC) and BMD were measured by using DEXA.

Waist and hip circumferences were also measured with an anthropometric tape while the participants were wearing light clothing. Waist circumference (WC) was measured at the minimum circumference between the iliac crest and the rib cage. Hip circumference (HC) was measured at the maximum protuberance of the buttocks. These measurements were done in duplicate. Blood pressure was measured by using an Omron blood pressure monitor (model HEM-907). Hand grip strength (HGS) was measured by using hand dynamometer (Lafayette Instrument, USA). Measurements were taken in duplicate and readings were averaged.

### Measurement of 25OH-D_3_ by LC-MS/MS

Since vitamin D_3_ is the most utilized form of vitamin D in clinical trials and it accounts for a major fraction of the circulating 25OH-D, only 25OH-D_3_ results will be discussed in this study. In the following, vitamin D refers to 25OH-D_3_ unless specified otherwise.

Plasma samples were analyzed using Waters Xevo TQ-S LC-MS/MS system equipped with a Waters ACQUITY UPLC (Waters Corporation, MA, U.S.A.). A Waters ACQUITY UPLC CSH Phenyl-Hexyl column (2.1 × 100 mm, 1.7 μm) at 35°C was used and the flow rate was 0.4 mL/min. Electro spray ionization (ESI) in the positive ion mode and multiple reaction monitoring (MRM) mode were used for LC-MS/MS. The respective transitions at *m/z* 383.35→ *m/z* 107.15, 257.25 and *m/z* 389.35→ *m/z* 263.25, 107.15 for 25OH-D_3_ and 25OH-D_3_ d_6_ were monitored. The auto dwells were used for MRM. The desolvation gas was nitrogen (1000 L/h) and the collision gas was Argon (10 psi), respectively. The capillary voltage was set at 3.2 kV, and the desolvation temperature was maintained at 550°C.

### Statistical analysis

Baseline characteristics of the participants were presented as arithmetic means ± SDs. Linear regression models were used to examine the associations between vitamin D status and various clinical measures. Age was controlled for in the regression models whereby the clinical measures of participants without vitamin D deficiency (*n* = 66) was compared with those with vitamin D deficiency (*n* = 48). Vitamin D deficiency is defined as individuals with vitamin D concentrations < 20 ng/mL [30]. In addition, gender was controlled for in the regression models examining the associations between vitamin D concentrations and body fat percentage. All statistical analyses were performed using Stata 11.1 (StataCorp, College Station, Tex, USA). Two sided *p* < 0.05 was considered statistically significant in all cases.

## Results

**Fig 1:**
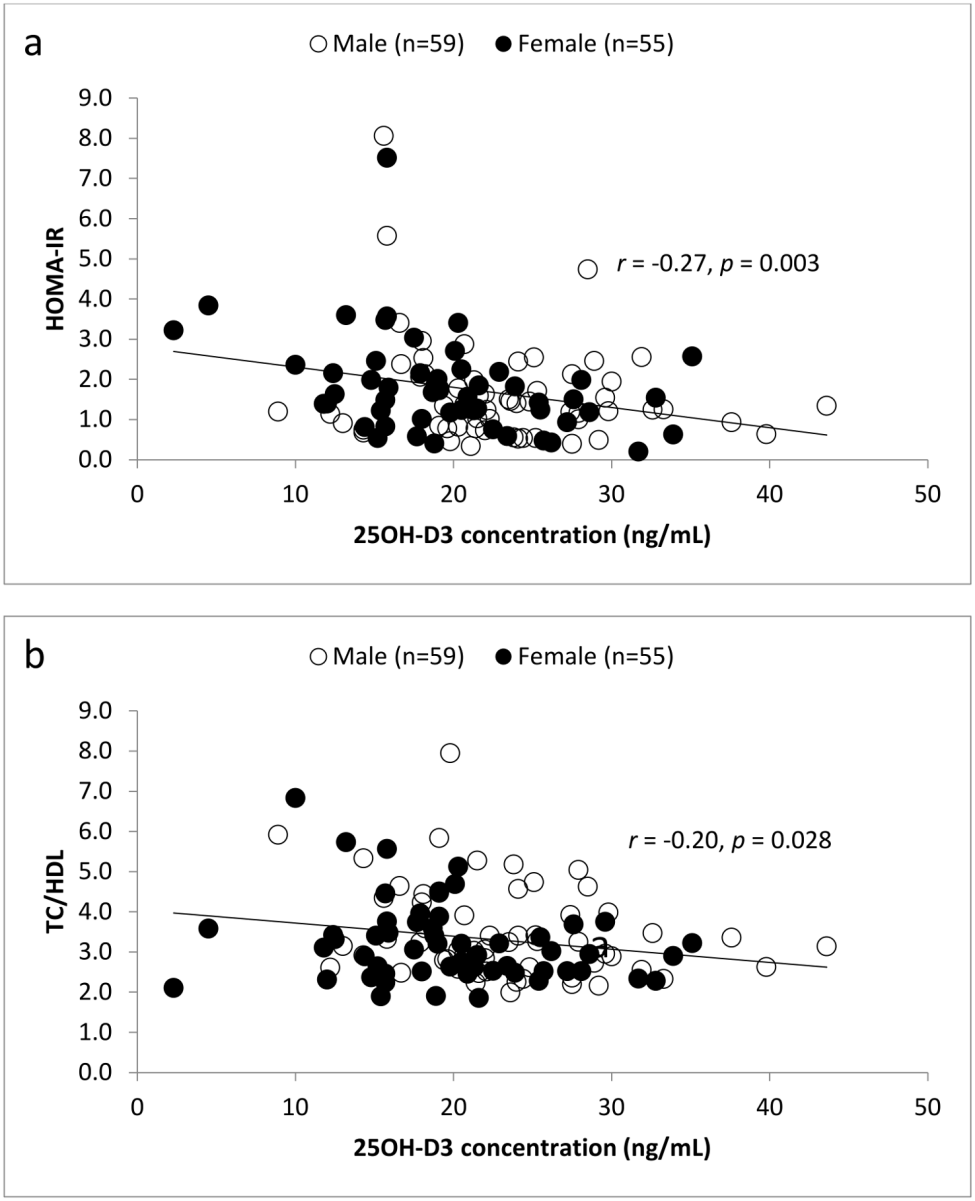
Relation between vitamin D concentrations and (a) HOMA-IR, and (b) TC/HDL ratio.

The clinical characteristics of the study population are summarized in Table 1. Generally, males had higher 25OH-D_3_ concentrations than females. In our study, 54.5% of females had 25OH-D_3_ less than 20 ng/mL (vitamin D deficiency), whereas 30.5% of males were classified as vitamin D deficient. Four participants, including one male and three females, had 25OH-D_3_ levels ≤ 10 ng/mL. As shown in Table 2, compared with participants without vitamin D deficiency, 25OH-D_3_ deficient participants were significantly shorter (~ 6 cm) and had greater BMI (~ 1.5 kg/m^2^). Moreover, they had lower HGS (~ 5 kg), but higher FSI (~ 3 mU/L), HOMA-IR (~ 0.7), LDL (~ 0.5 mmol/L), TC/HDL ratio (~ 0.5), and LDL/HDL ratio (~ 0.4) than those without vitamin D deficiency. Results remained statistically significant after adjusting for age, except for BMI (from *p* = 0.047 to *p* = 0.079) and LDL (from *p* = 0.046 to *p* = 0.057) whereby the associations were attenuated. The significant inverse correlations between vitamin D status, HOMA-IR, and TC/HDL ratio were also shown in Fig 1. On the other hand, no associations were found between vitamin D status and age, weight, WC, waist-to-hip ratio (WHR), blood pressure (BP), BMC, BMD, FBG, TG, TC, and HDL. In this study, three measuring techniques, including BIA, BOD POD and whole body DEXA scans, were employed to measure body fat percentage and LBM. Regardless of the measuring techniques, vitamin D deficient participants had a significantly higher body fat percentage and a lower LBM than those without deficiency.

**Table 1:** Characteristics of the study population.

	Total (*n*=114)	Male (*n*=59)	Female (*n*=55)
Age (y)	31.5 ± 12.4	30.9 ± 11.9	32.2 ± 13.0
Height (cm)	166.6 ± 9.1	172.3 ± 6.7	160.5 ± 7.2
Weight (kg)	63.7 ± 13.9	70.1 ± 11.7	56.8 ± 12.7
BMI (kg/m^2^)	22.8 ± 3.9	23.6 ± 3.8	21.9 ± 3.9
WC (cm)	74.4 ± 10.3	78.4 ± 9.6	70.1 ± 9.4
WHR	0.81 ± 0.07	0.85 ± 0.05	0.77 ± 0.05
Body fat (%)	25.1 ± 9.6	19.8 ± 7.9	30.7 ± 7.9
LBM (kg)	47.5 ± 10.7	55.7 ± 6.6	38.7 ± 6.4
BMC (kg)	2.25 ± 0.46	2.54 ± 0.34	1.94 ± 0.35
BMD (g/cm^2^)	1.13 ± 0.11	1.18 ± 0.09	1.07 ± 0.09
Hip BMD (g/cm^2^)	0.96 ± 0.14	1.03 ± 0.14	0.89 ± 0.12
HGS (left) (kg)	27.7 ± 9.3	34.8 ± 6.3	20.2 ± 4.9
HGS (right) (kg)	29.3 ± 9.5	36.8 ± 6.1	21.3 ± 4.7
FBG (mmol/L)	4.6 ± 0.5	4.7 ± 0.5	4.5 ± 0.5
FSI (mU/L)	8.2 ± 5.4	7.8 ± 5.7	8.7 ± 5.0
HOMA-IR	1.7 ± 1.3	1.6 ± 1.3	1.8 ± 1.2
TG (mmol/L)	0.86 ± 0.45	0.88 ± 0.39	0.85 ± 0.51
TC (mmol/L)	5.1 ± 1.5	5.0 ± 1.8	5.2 ± 1.1
HDL (mmol/L)	1.6 ± 0.4	1.5 ± 0.3	1.7 ± 0.4
LDL (mmol/L)	3.1 ± 1.3	3.1 ± 1.6	3.1 ± 0.9
TC/HDL	3.3 ± 1.1	3.5 ± 1.2	3.2 ± 1.0
LDL/HDL	2.1 ± 0.9	2.2 ± 1.0	2.0 ± 0.8
25OH-D_3_ (ng/mL)	21.6 ± 6.9	23.3 ± 6.6	19.8 ± 6.7

Abbreviations: BMI, body mass index; WC, waist circumference; WHR, waist-to-hip ratio; LBM, lean body mass; BMC, bone mineral content; BMD, bone mineral density; HGS, hand grip strength; FBG, fasting blood glucose; FSI, fasting serum insulin; HOMA-IR, homeostasis model assessment of insulin resistance; TG, triglycerides; TC, total cholesterol; HDL, high-density lipoprotein; LDL, low-density lipoprotein.

Values are expressed as mean ± SD.

Percent body fat and LBM were measured using BOD POD.

BMC and BMD were measured using DEXA.

To convert 25OH-D_3_ concentrations to nanomoles per liter, multiply by 2.5.

**Table 2:** Clinical measures of 114 participants separated by vitamin D deficiency status.

	Without deficiency (*n*=66)	Deficiency (*n*=48)	*p*-value
Age (y)	30.5 ± 11.4	33.0 ± 13.6	0.292
Height (cm)	169.0 ± 8.9	163.4 ± 8.4	0.001
Weight (kg)	63.7 ± 11.5	63.7 ± 16.7	0.984
BMI (kg/m^2^)	22.2 ± 3.1	23.7 ± 4.8	0.047
WC (cm)	73.7 ± 8.6	75.4 ± 12.3	0.378
WHR	0.81 ± 0.06	0.82 ± 0.07	0.703
Body fat (%)^a^	21.2 ± 7.4	27.7 ± 8.8	<0.001
Body fat (%)^b^	22.3 ± 8.8	28.8 ± 9.4	<0.001
Body fat (%)^c^	26.7 ± 7.8	32.7 ± 7.7	<0.001
LBM (kg)^d^	49.5 ± 10.6	44.7 ± 10.3	0.017
LBM (kg)^e^	46.6 ± 10.2	42.4 ± 10.9	0.041
SBP (mm Hg)	111.8 ± 13.4	112.4 ± 18.9	0.832
DBP (mm Hg)	66.2 ± 8.7	65.1 ± 13.1	0.602
BMC (kg)	2.30 ± 0.45	2.19 ± 0.47	0.210
BMD (g/cm^2^)	1.13 ± 0.11	1.12 ± 0.11	0.571
Hip BMD (g/cm^2^)	0.97 ± 0.14	0.96 ± 0.15	0.687
HGS (left) (kg)	29.9 ± 9.0	24.8 ± 9.0	0.004
HGS (right) (kg)	31.5 ± 8.8	26.2 ± 9.7	0.003
FBG (mmol/L)	4.6 ± 0.5	4.7 ± 0.5	0.311
FSI (mU/L)	7.0 ± 4.0	9.9 ± 6.6	0.005
HOMA-IR	1.4 ± 0.8	2.1 ± 1.6	0.004
TG (mmol/L)	0.81 ± 0.31	0.94 ± 0.59	0.125
TC (mmol/L)	4.9 ± 1.0	5.3 ± 2.0	0.108
HDL (mmol/L)	1.6 ± 0.4	1.5 ± 0.3	0.081
LDL (mmol/L)	2.9 ± 0.8	3.4 ± 1.7	0.046
TC/HDL	3.1 ± 0.8	3.7 ± 1.3	0.005
LDL/HDL	1.9 ± 0.7	2.3 ± 1.1	0.006
25OH-D_3_ (ng/mL)	26.0 ± 5.1	15.6 ± 3.7	<0.001

Abbreviations: BMI, body mass index; WC, waist circumference; WHR, waist-to-hip ratio; LBM, lean body mass; SBP, systolic blood pressure; DBP, diastolic blood pressure; BMC, bone mineral content; BMD, bone mineral density; HGS, hand grip strength; FBG, fasting blood glucose; FSI, fasting serum insulin; HOMA-IR, homeostasis model assessment of insulin resistance; TG, triglycerides; TC, total cholesterol; HDL, high-density lipoprotein; LDL, low-density lipoprotein.

Values are expressed as mean ± SD.

Percent body fat was measured by ^a^BIA, ^b^BOD POD, and ^c^DEXA, respectively.

LBM was measured by ^d^BODPOD and ^e^DEXA, respectively.

BMC and BMD were measured by DEXA.

To convert 25OH-D_3_ concentrations to nanomoles per liter, multiply by 2.5.

A 25OH-D_3_ concentration < 20 ng/mL is an indication of vitamin D deficiency.

Table 3 shows the mean values for 25OH-D_3_ concentrations in participants with body fat percentage < 25% and those with body fat percentage ≥ 25%, respectively. Whereas mean values were significantly lower in females than males (19.8 vs. 23.3 ng/mL; *p* = 0.006), gender differences in vitamin D concentrations did not persist after adjusting for body fat percentage (*p* = 0.248). In addition, there was no evidence of statistically significant difference in 25OH-D_3_ concentrations between females and males after the results being separated by body fat percentage (both *p* ≥ 0.098).

**Table 3:** 25OH-D_3_ concentrations of 114 participants separated by gender and body fat (%).

	25OH-D_3_ (ng/mL)			
	All (n=114)	Male (*n*=59)	Female (*n*=55)	*p*-value^a^
All body fat (%) (*n*=114)	21.6 ± 6.9	23.3 ± 6.6	19.8 ± 6.7	0.006
Body fat (%) <25% (*n*=59)	23.6 ± 6.6^b^	24.4 ± 6.3	21.2 ± 7.0	0.098
Body fat (%) ≥25% (*n*=55)	19.5 ± 6.5	20.0 ± 6.4	19.3 ± 6.6	0.740

Values are expressed as mean ± SD.

^a^ Values between males and females were compared by using regression models.

^b^ Indicates a significant difference between participants with low and high body fat (%) (*p* = 0.001).

Percent body fat was measured using BOD POD.

To convert 25OH-D_3_ concentrations to nanomoles per liter, multiply by 2.5.

When male and female participants were analyzed independently, significant differences were observed between vitamin D status and HOMA-IR for both male (*p* = 0.042) and female (*p* = 0.054) participants (Table 4). However, no significant difference was observed between vitamin D levels and TC/HDL ratio for females. In contrast, there was a significant inverse correlation between 25OH-D_3_ levels, WHR (*r* = -0.28, *p* = 0.034), and TC/HDL (*r* = -0.27, *p* = 0.041) in male participants.

**Table 4:** Clinical measures of 59 males and 55 females separated by vitamin D deficiency status.

	Male			Female		
	Without deficiency (*n*=41)	Deficiency (*n*=18)	*p*-value	Without deficiency (*n*=25)	Deficiency (*n*=30)	*p*-value
Age (y)	30.5 ± 11.1	31.9 ± 13.9	0.683	30.5 ± 12.2	33.6 ± 13.7	0.377
Height (cm)	173.3 ± 6.9	170.0 ± 5.8	0.077	161.9 ± 7.2	159.4 ± 7.2	0.211
Weight (kg)	69.1 ± 9.2	72.5 ± 16.1	0.314	54.7 ± 9.0	58.5 ± 15.0	0.282
BMI (kg/m^2^)	23.0 ± 2.9	25.0 ± 5.1	0.059	20.8 ± 2.9	22.8 ± 4.5	0.060
WC (cm)	76.9 ± 7.9	81.9 ± 12.2	0.062	68.5 ± 7.2	71.5 ± 10.8	0.234
WHR	0.84 ± 0.05	0.87 ± 0.06	0.034	0.76 ± 0.05	0.78 ± 0.06	0.210
Body fat (%)^a^	17.4 ± 5.2	21.3 ± 6.5	0.017	27.3 ± 6.4	31.5 ± 7.7	0.033
Body fat (%)^b^	18.1 ± 7.1	23.6 ± 8.4	0.013	29.2 ± 6.8	31.9 ± 8.6	0.209
Body fat (%)^c^	22.3 ± 5.3	26.6 ± 5.9	0.007	33.8 ± 5.5	36.5 ± 6.2	0.097
LBM (kg)^d^	56.3 ± 6.4	54.3 ± 7.1	0.293	38.4 ± 5.4	38.9 ± 7.1	0.746
LBM (kg)^e^	53.3 ± 6.1	52.3 ± 8.3	0.615	35.9 ± 5.1	36.3 ± 7.3	0.823
SBP (mm Hg)	118.0 ± 11.5	116.2 ± 7.2	0.526	101.5 ± 9.4	110.2 ± 23.1	0.084
DBP (mm Hg)	68.2 ± 9.2	62.4 ± 15.2	0.077	62.8 ± 6.7	66.7 ± 11.6	0.147
BMC (kg)	2.52 ± 0.36	2.59 ± 0.29	0.519	1.93 ± 0.32	1.94 ± 0.39	0.971
BMD (g/cm^2^)	1.17 ± 0.10	1.20 ± 0.07	0.291	1.06 ± 0.09	1.07 ± 0.10	0.839
Hip BMD (g/cm^2^)	1.01 ± 0.14	1.05 ± 0.12	0.298	0.89 ± 0.11	0.89 ± 0.13	0.950
HGS (left) (kg)	35.2 ± 6.3	33.9 ± 6.6	0.490	21.1 ± 5.1	19.4 ± 4.7	0.195
HGS (right) (kg)	37.0 ± 6.0	36.3 ± 6.5	0.716	22.6 ± 3.8	20.2 ± 5.2	0.057
FBG (mmol/L)	4.6 ± 0.5	4.8 ± 0.5	0.180	4.5 ± 0.4	4.6 ± 0.6	0.560
FSI (mU/L)	6.9 ± 4.1	9.8 ± 8.1	0.073	7.2 ± 3.8	9.9 ± 5.6	0.048
HOMA-IR	1.4 ± 0.8	2.2 ± 1.9	0.042	1.5 ± 0.8	2.1 ± 1.4	0.054
TG (mmol/L)	0.84 ± 0.33	0.97 ± 0.50	0.238	0.76 ± 0.28	0.92 ± 0.64	0.238
TC (mmol/L)	4.7 ± 0.9	5.6 ± 2.9	0.060	5.2 ± 1.1	5.1 ± 1.1	0.895
HDL (mmol/L)	1.5 ± 0.3	1.4 ± 0.3	0.130	1.8 ± 0.4	1.6 ± 0.3	0.030
LDL (mmol/L)	2.8 ± 0.8	3.8 ± 2.5	0.029	3.0 ± 0.9	3.1 ± 1.0	0.669
TC/HDL	3.2 ± 0.9	4.1 ± 1.4	0.004	3.0 ± 0.7	3.4 ± 1.1	0.099
LDL/HDL	1.9 ± 0.8	2.7 ± 1.3	0.004	1.8 ± 0.6	2.1 ± 0.9	0.107
25OH-D_3_ (ng/mL)	26.3 ± 5.4	16.5 ± 3.0	<0.001	25.5 ± 4.5	15.1 ± 4.1	<0.001

Abbreviations: BMI, body mass index; WC, waist circumference; WHR, waist-to-hip ratio; LBM, lean body mass; SBP, systolic blood pressure; DBP, diastolic blood pressure; BMC, bone mineral content; BMD, bone mineral density; HGS, hand grip strength; FBG, fasting blood glucose; FSI, fasting serum insulin; HOMA-IR, homeostasis model assessment of insulin resistance; TG, triglycerides; TC, total cholesterol; HDL, high-density lipoprotein; LDL, low-density lipoprotein.

Values are expressed as mean ± SD.

Percent body fat was measured by ^a^BIA, ^b^BOD POD, and ^c^DEXA, respectively.

Lean body mass was measured by ^d^BODPOD and ^e^DEXA, respectively.

BMC and BMD were measured by DEXA.

To convert 25OH-D_3_ concentrations to nanomoles per liter, multiply by 2.5.

A 25OH-D_3_ concentration < 20 ng/mL is an indication of vitamin D deficiency.

## Discussion

While it has been reported that living at higher latitudes correlated with high risk of vitamin D deficiency, we found that 42.1% of participants living in Singapore (1°18'N 103°51'E with the mean sunshine of 2022.4 h/y) were at risk of vitamin D deficiency (< 20 ng/mL). The high prevalence of vitamin D deficiency among people residing in a tropical country is of concern. Previously, similar results were observed in Saudi adults, whereby 52.5% of participants were either deficient or insufficient of vitamin D. Vitamin D deficiency was more pronounced in females, which was partly attributed to wearing of traditional clothes (i.e. a black outer cloak and a gauzy scarf) [31]. In our study, females (54.5%) had a higher prevalence of vitamin D deficiency than males (30.5%), although Singaporean females do not wear outer cloak and scarf. Among the participants studied, two females had a severe vitamin D deficiency (< 5 ng/mL). One of the possible reasons is that females are much more frequent users of sunscreen than males, which lowers the amount of sun exposure and thus vitamin D synthesis. The second possible reason is that females had a higher body fat percentage than males. The low vitamin D status in female participants is probably a consequence of an increased sequestration of vitamin D in adipose tissue [6]. We found that the difference of vitamin D deficiency between males and females did not persist after adjusting for body fat percentage, suggesting that body fat may be one of the predominant risk factors for vitamin D deficiency.

In view of the prevalence of vitamin D deficiency and obesity, it is possible that vitamin D status is an independent predictor of the increased body fat. To accurately and independently assess the body fat-vitamin D relationship, three fat tissue measuring techniques were used in the current study. All three measuring techniques consistently demonstrate an inverse association between circulating vitamin D and degree of adiposity as defined by BMI and body fat percentage. Thus, obesity may, in part, be caused by vitamin D deficiency and/or vice versa. In addition, we found that the association of body fat percentage (*r* = -0.30, *p* = 0.001) with vitamin D status was more pronounced than that of BMI (*r* = -0.17, *p* = 0.074), suggesting that body fat percentage represents a more powerful predictor of vitamin D status compared to BMI. Previous studies reported that low vitamin D status was more obvious in adults with a BMI of 30 kg/m^2^ or more [6]. Participants in the present study have relatively low BMI values with mean BMI of 23.6 kg/m^2^ for males and 21.9 kg/m^2^ for females. Therefore, the association between vitamin D levels and BMI is not as significant as the body fat percentage. In addition, we found a positive relationship of vitamin D levels with LBM (*r* = 0.24, *p* = 0.010). Overall these results suggest that future studies which investigate vitamin D status in Asian population with lean body shape should utilize body fat percentage or LBM instead of BMI.

Although previous studies reported that vitamin D supplementation improved BMD and reduced the risk of osteoporosis and fractures [7, 8], the present study failed to confirm such a relationship. This is probably because the vitamin D-BMD relationship was complex. For example, this relationship appears to vary by race, being weaker in African-American or Hispanic ethnicity than in White population [11]. Another hypothesis is that the negative effects of vitamin D deficiency on bone mass or density may not be present in healthy adults.

It is known that obesity, especially central obesity, is an important risk factor for type 2 diabetes mellitus (T2DM). We observed an inverse relationship of vitamin D levels with WHR in male participants, but not females. The reason for the gender-difference in correlation with vitamin D status was not completely understood, probably associated with the level of sex hormones such as estrogen [32]. The negative impacts of WHR on vitamin D levels indicated the specific role of abdominal fat tissue on vitamin D status. Since WHR is a good predictor of T2DM, the risk of vitamin D deficiency should be monitored in males with higher fat mass, lower LBM, and greater WHR.

Like obesity, vitamin D deficiency has become a global epidemic and a risk factor for T2DM, which is characterized by insulin resistance and altered insulin secretion. Vitamin D has been proposed to play an important role in the development of insulin resistance and the pathogenesis of T2DM by affecting either insulin sensitivity of beta-cell function [33]. The molecular actions of vitamin D on insulin resistance included gene polymorphisms, immunoregulatory function, and inflammation [34–36]. Our study indicated that vitamin D deficiency increased FSI and HOMA-IR in Asians, but not FBG. However, studies in European elderly yielded contrary results showing an inverse association of vitamin D status with FBG, whereas no relation with FSI and HOMA-IR [37]. Differences in study results can be partly explained by differences in participant characteristics, i.e. the participants in our study were relatively young Asians. The large-scale population studies, such as NHANES III data, have shown a significant relationship between vitamin D and HOMA-IR in Caucasian and Mexican Americans, but not in African Americans [38].

Accumulating research suggests that vitamin D status may be associated with dyslipidemia and thus cardiovascular disease (CVD). Epidemiological studies have shown that atherogenic dyslipidemia (AD), referring to elevated levels of TG and LDL and decreased levels of HDL, is an important independent risk factor for CVD. However, the associations between vitamin D and lipid profile are divergent. While some studies have reported a positive association between vitamin D status and HDL [39], others, like ours, found no such relation [40]. It should be noted that in our study, when male and female participants were analyzed independently, a positive correlation between vitamin D levels and HDL was observed in female participants, whereas an inverse correlation between vitamin D levels and LDL was observed in male participants. The gender-difference in correlation with vitamin D status should be further studied by increasing the sample size. Previous research suggests that the ratio of TC/HDL is more useful than individual parameter in predicting cardiometabolic risk [41]. For example, TC > 6.2 mmol/L (240 mg/dL) is a risk factor for CVD, but the risk is lower if a high proportion is made up of the protective HDL than if the elevation is due primarily to increased amounts of the atherogenic LDL. The present study shows significant inverse associations between vitamin D status, TC/HDL, and LDL/HDL, suggesting that vitamin D deficiency may serve as a potential predictor for CVD.

In conclusion, the high prevalence of vitamin D deficiency in a population living in the sunny tropical region is of concern. Our study supports the hypothesis that obesity (the increased body fat) is a risk factor for vitamin D deficiency or vitamin D deficiency is a risk factor for obesity. The positive associations between vitamin D status, LBM, and HGS indicate that vitamin D is necessary for strong muscles. However, our data does not show any association between bone health and vitamin D status. Additionally, vitamin D status is inversely associated with HOMA-IR and TC/HDL ratio. Because our study was cross-sectional, the cause-and-effect relationships between vitamin D status, HOMA-IR, and TC/HDL cannot be established. Future randomized controlled trials are needed to evaluate whether low vitamin D levels are associated with T2DM and CVD and to determine possible mechanisms of any preventive effect from vitamin D intake against T2DM and CVD. Despite the limitations, the clinical relevance of our findings is important. Our results add to existing knowledge by identifying vitamin D deficiency as a potential risk factor for cardiovascular risks including insulin resistance, AD, hypertension, and glucose intolerance.
